# Left–Right Asymmetry in Spectral Characteristics of Lung Sounds Detected Using a Dual-Channel Auscultation System in Healthy Young Adults

**DOI:** 10.3390/s17061323

**Published:** 2017-06-07

**Authors:** Jang-Zern Tsai, Ming-Lang Chang, Jiun-Yue Yang, Dar Kuo, Ching-Hsiung Lin, Cheng-Deng Kuo

**Affiliations:** 1 Department of Electrical Engineering, National Central University, Zhongli, Taiyuan 320, Taiwan; jztsai@ee.ncu.edu.tw (J.-Z.T.); alan.chang@si-xpandas.com (M.-L.C.); jim@ytec.com.tw (J.-Y.Y.); 2 Mt. San Antonio College, Walnut, CA 91789, USA; dkuo11@student.mtsac.edu; 3 Department of Respiratory Care, College of Health Sciences, Chang Jung Christian University, Tainan 711, Taiwan; teddy@cch.org.tw; 4 School of Medicine, Chung Shan Medical University, Taichung 402, Taiwan; 5 Division of Chest Medicine, Department of Internal Medicine, Changhua Christian Hospital, Changhua 500, Taiwan; 6 Laboratory of Biophysics, Department of Medical Research, Taipei Veterans General Hospital, Taipei 112, Taiwan

**Keywords:** auscultation, lung sounds, stethoscope, power spectrum, dual-channel

## Abstract

Though lung sounds auscultation is important for the diagnosis and monitoring of lung diseases, the spectral characteristics of lung sounds have not been fully understood. This study compared the spectral characteristics of lung sounds between the right and left lungs and between healthy male and female subjects using a dual-channel auscultation system. Forty-two subjects aged 18–22 years without smoking habits and any known pulmonary diseases participated in this study. The lung sounds were recorded from seven pairs of auscultation sites on the chest wall simultaneously. We found that in four out of seven auscultation pairs, the lung sounds from the left lung had a higher total power (P_T_) than those from the right lung. The P_T_ of male subjects was higher than that of female ones in most auscultation pairs. The ratio of inspiration power to expiration power (R_I/E_) of lung sounds from the right lung was greater than that from the left lung at auscultation pairs on the anterior chest wall, while this phenomenon was reversed at auscultation pairs on the posterior chest wall in combined subjects, and similarly in both male and female subjects. Though the frequency corresponding to maximum power density of lung sounds (F_MPD_) from the left and right lungs was not significantly different, the frequency that equally divided the power spectrum of lung sounds (F_50_) from the left lung was significantly smaller than that from the right lung at auscultation site on the anterior and lateral chest walls, while it was significantly larger than that of from the right lung at auscultation site on the posterior chest walls. In conclusion, significant differences in the P_T_, F_MPD_, F_50_, and R_I/E_ between the left and right lungs at some auscultation pairs were observed by using a dual-channel auscultation system in this study. Structural differences between the left and the right lungs, between the female and male subjects, and between anterior and posterior lungs might account for the observed differences in the spectral characteristics of lung sounds. The dual-channel auscultation system might be useful for future development of digital stethoscopes and power spectral analysis of lung sounds in patients with various kinds of cardiopulmonary diseases.

## 1. Introduction

Respiration rate is one of the vital signs used daily in clinical settings including anesthesiology and critical care medicine, in addition to body temperature, pulse rate, and blood pressure. Not only respiration rate but also the pattern of respiration and the pitches and loudness of lung sounds heard during respiration are important for the diagnosis and monitoring of cardiopulmonary diseases. Thus, the auscultation of lung sounds is one of the clinical practices of physicians in the diagnosis and treatment of asthma, chronic bronchitis, chronic obstructive pulmonary disease (COPD), bronchiectasis, pulmonary edema, acute respiratory distress syndrome (ARDS), etc. The rhonchi, rale, wheezing, and stridor heard during auscultation are important clues for the diagnosis of secretion accumulation in the lung, pulmonary edema, asthma, upper airway obstruction, etc. 

The stethoscope has been one of the most frequently used medical diagnostic tools in clinical settings. Its low price, simple structure, portability, safety, noninvasiveness, and quick diagnosis have enabled it to survive for more than a century as a medical device. Moreover, it has become an icon of physicians. Electronic stethoscopes with higher sensitivity have been introduced as technology advances [1]. With the ability to store and manipulate every bit of data, digital technology can bring more impact to auscultation. By using digital stethoscopes, not only the processing of acquired signals but also the way of auscultation has become versatile.

The lung sounds are caused by the turbulence of airflow in the respiratory tracts. Spectrum of lung sounds is mainly distributed from 60 to 600 Hz [2]. Lung sounds recorded from the chest wall include muscular and alveolar sounds [3]. The amplitude of lung sounds can be different from one location to another. The frequency distribution of lung sound signals changes with the age of the subject [4]. By using linear regression analysis to analyze the relation between age and the ratio (Q) of the powers of two frequency bands, from 330 to 600 Hz and from 60 to 330 Hz, it was found that there was a significant correlation between the age and the Q [5]. Lung sounds and tracheal sound are important parameters in clinical diagnosis of diseases. Experiments have shown that the temporal variability of tracheal sounds is bigger than that of lung sounds and that the spectral pattern of tracheal and lung sounds are stable with low intra-subject variability [6]. Further, the body height can make a difference in the length of trachea and hence a difference in tracheal sound [7].

The researches on the lung sounds have lasted for decades. Nairn and Turner-Warwick [8] used radioactive rays to scan the lung and found that the airflow inside the lung had a highly positive correlation with lung sound amplitude. Leblanc [9] and Polysongsang [10,11] also used radioactive rays to scan the lung, and found that the volume inhaled inside the lungs has a highly positive correlation with the lung sound amplitude. Kompis et al. [12] recorded the lung sounds simultaneously with 16 microphones distributed over the thoracic surface, and found that inspiratory sounds were 10–11 dB louder than expiratory sounds at comparable flow rates, and that for the front part of the thorax, the lung sounds were louder on the right side, and opposite for the back part. The same result was also shown in [13].

Though lung sound characteristics are important for the diagnosis and monitoring of lung diseases, the factors affecting the lung sound characteristics have not been fully understood. This study compared the spectral characteristics of lung sounds between paired auscultation sites on the left and right chest walls in healthy young adults by using a dual-channel auscultation system.

## 2. Methods

### 2.1. Lung Sounds Recording

**Figure 1:**
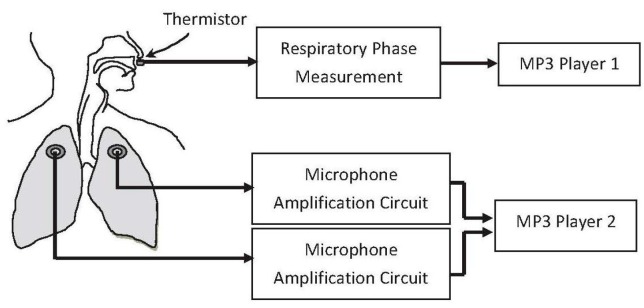
The hardware used to measure and record lung sounds and respiratory phase.

**Figure 2:**
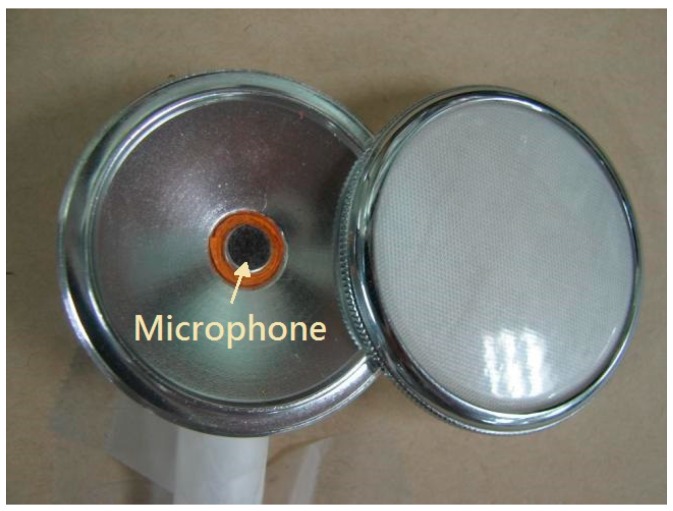
Each of the stethoscope head was made by encaving a condenser microphone into the bell taken from a conventional stethoscope.

**Figure 3:**
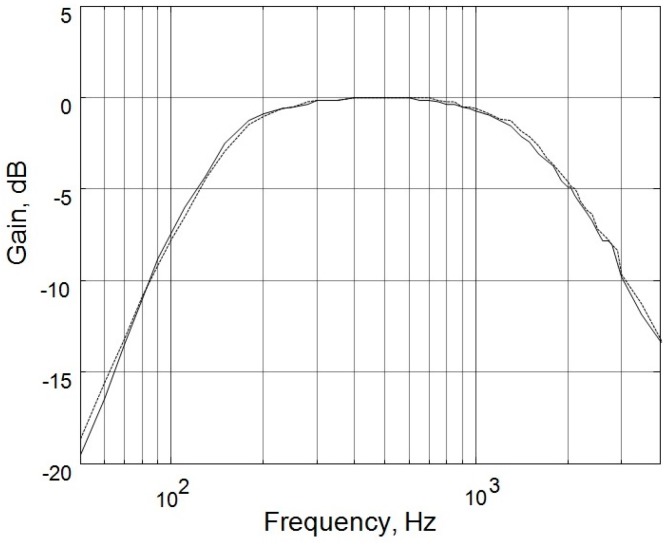
The normalized frequency response of the microphone amplification circuit. Solid line: left channel; dashed line: right channel.

Figure 1 shows the hardware of the dual-channel auscultation system used to record the lung sounds according to the breathing phase. The lung sounds were recorded in pairs with two condenser microphones embedded in a chest piece taken from a conventional stethoscope, as shown in Figure 2. The output signals of the microphones were amplified by the amplification circuits; both of which had a low cutoff frequency of 140 Hz, a high cutoff frequency of 1700 Hz, and a gain of 200. As shown in Figure 3, the two circuits had almost identical frequency responses. The differences in the two frequency responses were corrected computationally before the statistical analysis of lung sound characteristics. The paired amplified signals entered an MP3 player/recorder through the left and right channels at the microphone inputs of a personal computer and were sampled at 8 kHz and digitized into two sequences of 16-bit binary numbers, which were then stored in a flash memory as a data file.

### 2.2. Respiratory Phase Measurement 

The respiratory phase detecting circuit shown in Figure 1 measured the state of inspiration and expiration. It contained a thermistor, which was placed near the nostril orifice, where the temperature was somewhere between body temperature and room temperature. The impedance of the thermistor had a negative temperature coefficient. When the air was exhaled through the nostrils, the temperature around the thermistor rose so that the resistance of the thermistor decreased. Conversely, when the air was inhaled through the nostrils, the temperature around the thermistor fell so that the resistance of the thermistor increased. 

### 2.3. Subjects

Twenty-four male and eighteen female healthy young subjects recruited from the community were included in this study. Table 1 lists the basic data of the study subjects. All of them were nonsmokers, and none had any known cardiopulmonary diseases. For the purpose of statistical analysis, three subject groups were used, namely, the combined group, the male group, and the female group. The combined group included all male and female subjects recruited in this study. 

### 2.4. Sites of Auscultation

**Figure 4:**
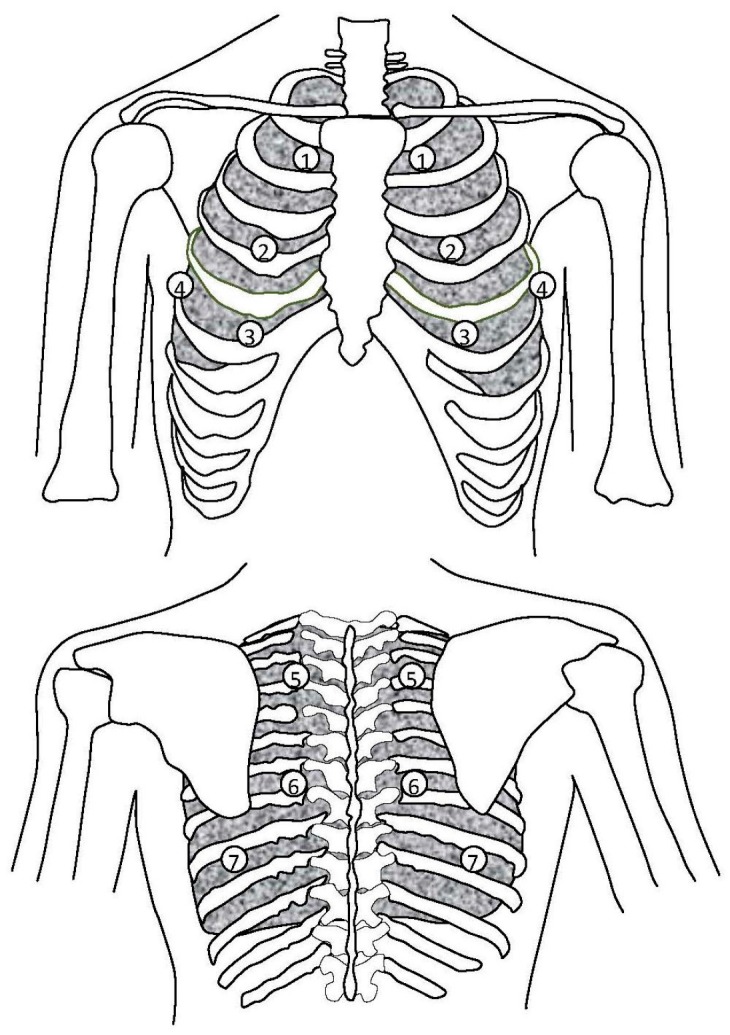
The auscultation sites over the chest walls. Auscultation pairs 1 to 3 were placed over the anterior chest wall; auscultation pair 4 was placed over the lateral chest wall; and auscultation pairs 5 to 7 were placed over the posterior chest wall. Auscultation pair 1 was placed between the 1st and the 2nd ribs. Auscultation pair 2 was placed between the 4th and the 5th ribs. Auscultation pair 3 was placed between the 6th and the 7th ribs. Auscultation pair 4 was placed between the 5th and the 6th ribs. Auscultation pair 5 was placed between the 2nd and the 3rd ribs. Auscultation pair 6 was placed between the 6th and the 7th ribs. Auscultation pair 7 was placed between the 8th and the 9th ribs.

Figure 4 shows the paired auscultation sites in this study. There were seven auscultation pairs, numbered from 1 to 7, representing the right and left auscultation sites at upper anterior, middle anterior, lower anterior, lateral, upper posterior, middle posterior, and lower posterior, respectively. Three pairs were on the anterior chest wall, one on the lateral wall, and the other three on the posterior chest wall. Each auscultation pair consisted of a left auscultation site and a right auscultation site. The lung sounds were recorded on the right and left sites of an auscultation pair simultaneously. 

### 2.5. Signal Processing and Analysis

The lung sound signals were analyzed by using the Matlab program (The MathWorks, Inc., Natick, MA, USA). Because the condenser microphone is very sensitive, noises due to movements of the tubes, rubbing of the stethoscope against the skin, and frictions between the stethoscope and the bandage were also recorded. These artifacts were removed by using a wavelet filter before spectral and statistical analyses.

### 2.6. Parameter Definitions

Fast Fourier Transform (FFT) was employed to analyze the stored lung sound signals in the frequency domain. The following four parameters were defined to characterize the spectral properties of the lung sounds:

P_T_ (total power): the integration of the power spectral density (PSD) over the entire frequency range;

R_I/E_ (inspiratory/expiratory power ratio): the ratio of the total power of lung sounds during inspiration to that during expiration;

F_50_ (50%-power frequency): the frequency at which the power accumulated from below is 50% of the total power;

F_MPD_ (maximum-power-density frequency): the frequency at which the power spectral density of lung sounds is the greatest.

### 2.7. Statistics

The quantile–quantile (Q-Q) plot was used to assess the distribution normality of P_T_, R_I/E_, F_50_, and F_MPD_ in the combined group, the male group, and the female group. The result indicated that they are not normally distributed. Hence, a nonparametric test, namely, the Wilcoxon signed rank test (SigmaPlot13 for Windows, Systat Software, Inc., San Jose, CA, USA) was used to compare the differences in the above-defined lung sound characteristics between the right and left lungs. Similarly, the Kruskal–Wallis rank sum test was employed to compare the differences in the lung sound characteristics between male and female subjects. Significance level was defined as *p* < 0.05. Linear regression analysis was used to disclose the relationship between the P_T_ of lung sounds and the body mass index (BMI) or the body height of the subjects. 

## 3. Results

### 3.1. P_T_: Left vs. Right

**Figure 5:**
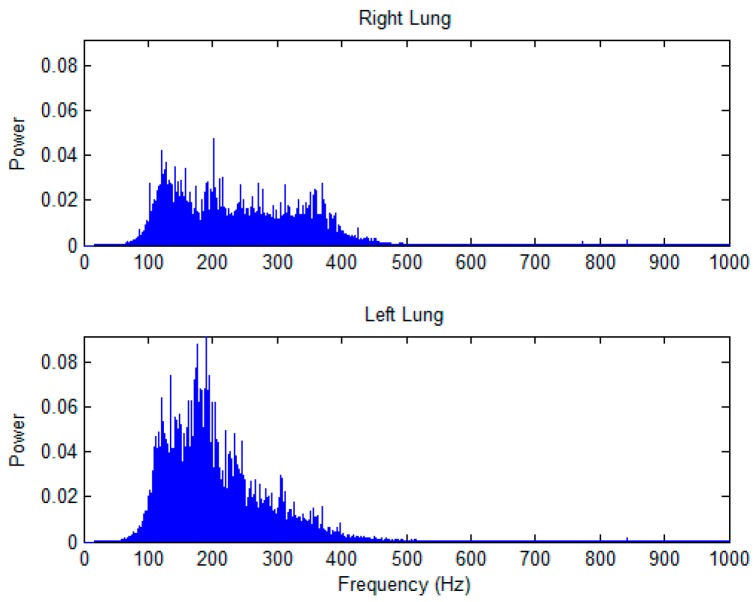
The power spectra of the right and left lungs of a female subject. Because the auscultation site of the left lung was close to the heart, the power in the low frequency range would be higher than that at the right lung.

**Figure 6:**
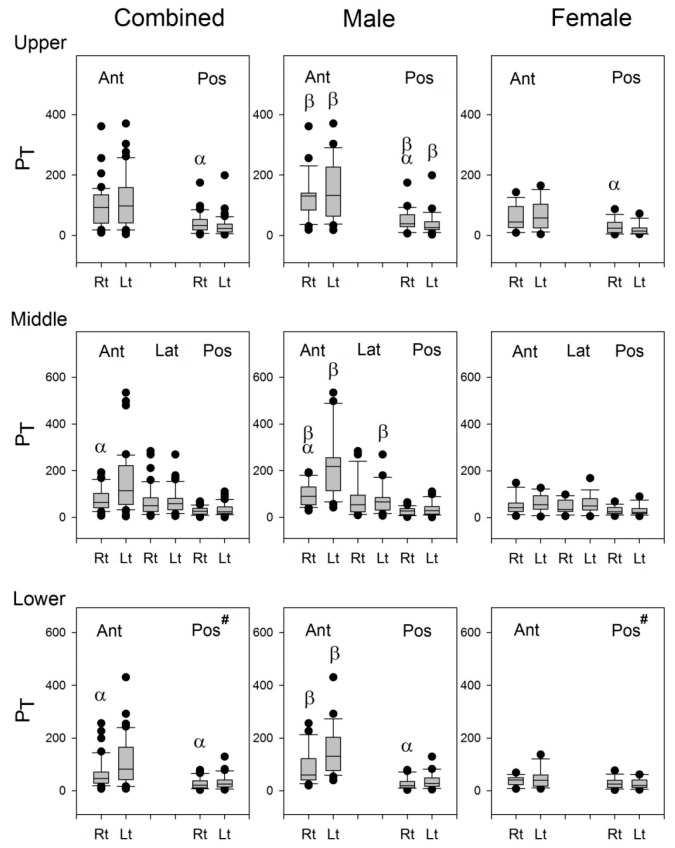
The boxplots of P_T_ in the combined group, male group, and female group. #: Three of the 18 female subjects were excluded from the statistics because of excessive artifacts at this site. α: *p* < 0.05 between right and left lungs, β: *p* < 0.05 vs. its counterpart in the female subjects. Ant: anterior; Pos: posterior; Lat: lateral; Rt: right, Lt: left.

A pair of typical power spectra of lung sounds recorded from one of the auscultation pairs are shown in Figure 5. As shown in Figure 6, the P_T_ of lung sounds recorded on the left lung was significantly higher than that on the right lung at Auscultation Pairs 2, 3, 5, and 7 in combined subjects, and at Auscultation Pairs 2, 3, 5, 6, and 7 in male subjects, except at Auscultation Pair 5 on the upper posterior chest wall where the P_T_ of lung sounds on the left lung was significantly smaller than that on the right lung. There was no significant difference in the P_T_ of lung sounds between left and right lungs at all auscultation pairs in female subjects, except at Auscultation Pair 5 where the P_T_ of lung sounds on the left lung was significantly smaller than that on the right lung.

### 3.2. P_T_: Male vs. Female

The characteristics of lung sounds were compared at the right and left auscultation sites between male and female subjects. A “β” on the boxplots in Figure 6 signifies a significant difference between the P_T_’s of the male and female subjects. The P_T_ of lung sounds of male subjects was higher than that of female subjects at Paired Auscultation Sites 1–5, except at the right lungs of Site 4. 

### 3.3. P_T_ vs. Body Height

It has been shown that body height can make a difference in the length of trachea, and hence a difference in tracheal sound [7]. Thus, body height might have an effect on the lung sound characteristic. We investigated the linear relation between body height and the P_T_ of lung sounds at various auscultation sites on the chest wall. Only the P_T_ of lung sounds at the upper anterior site of the right lung had significant linear relation with the body height of male subjects (Table 2). 

### 3.4. P_T_ vs. BMI

Table 3 shows the results of linear regression analysis between the BMI and the P_T_ of lung sounds at each auscultation site. The BMI of the male and female subjects were 23.6 ± 3.7 kg/m^2^ and 20.4 ± 1.7 kg/m^2^, respectively. Significant correlation between the P_T_ of lung sounds and BMI was found at the lower posterior site of the left lung in female subjects only. 

### 3.5. R_I/E_: Left vs. Right

**Figure 7:**
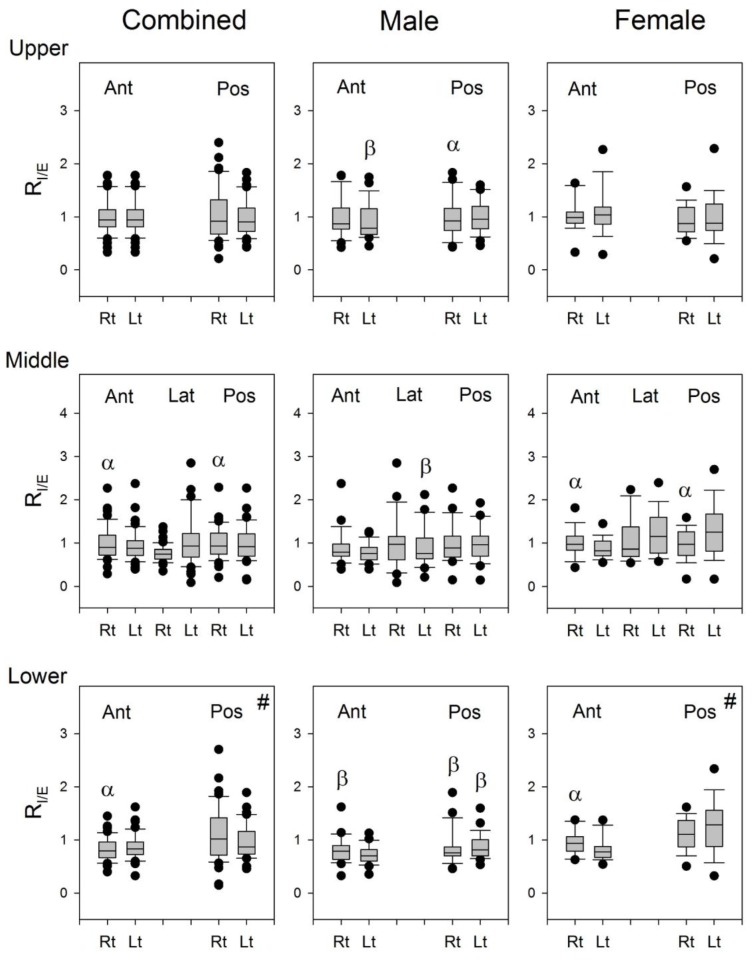
The boxplots of R_I/E_ in the combined group, male group, and female group. #: Three of the 18 female subjects were excluded from the statistics because of excessive artifacts at this site. α: *p* < 0.05 between right and left lungs, β: *p* < 0.05 vs. its counterpart in the female subjects. Ant: anterior; Pos: posterior; Lat: lateral; Rt: right, Lt: left.

The results of comparisons of the R_I/E_ between the right and left lungs in the combined group, the male group, and the female group, and between the male and female groups are shown in Figure 7. The R_I/E_ of lung sounds from the right lung was greater than that from the left lung at Auscultation Pairs 2 and 3 on the anterior chest wall, while it was smaller than that from the left lung at Auscultation Pair 6 on the posterior chest wall in the combined subjects. Similar results were found in the male and female subjects.

### 3.6. F_50_: Left vs. Right

**Figure 8:**
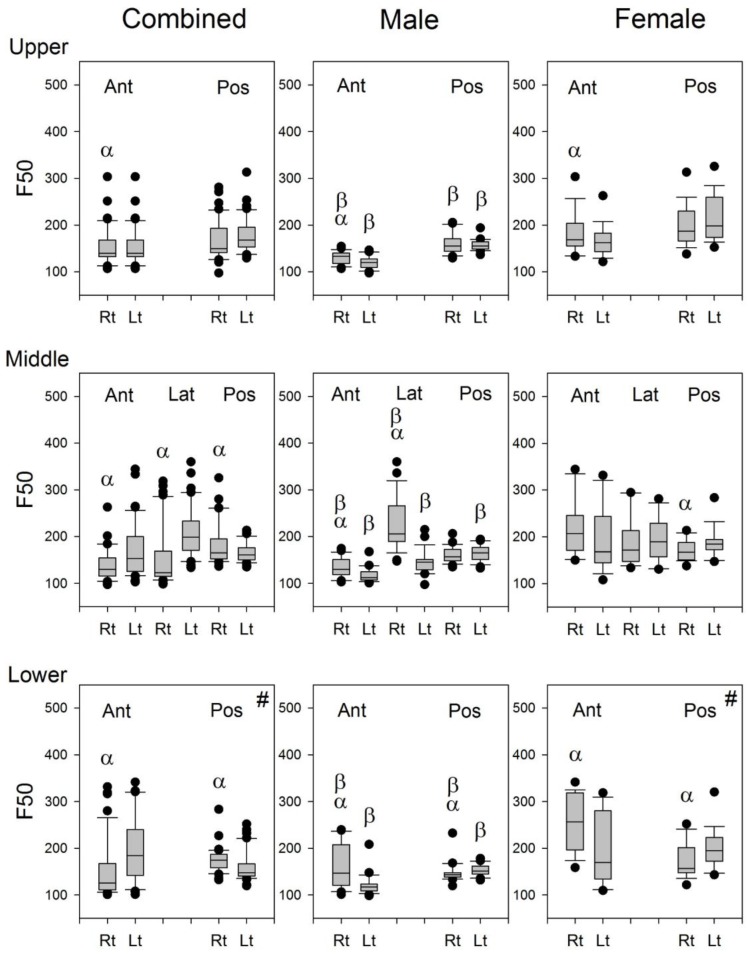
The boxplots of F_50_ in the combined group, male group, and female group. #: Three of the 18 female subjects were excluded from the statistics because of excessive artifacts at this site. α: *p* < 0.05 between right and left lungs; β: *p* < 0.05 vs. its counterpart in the female subjects. Ant: anterior; Pos: posterior; Lat: lateral; Rt: right, Lt: left.

The F_50_ of lung sounds was calculated and used as the representative frequency of that lung sounds. As shown in Figure 8, the F_50_ of the right lung was significantly greater than that of the left lung at Auscultation Pairs 1, 2, 3, and 4, while the F_50_ of the right lung was smaller than that at the left lung at Auscultation Pairs 6 and 7 for the combined subjects. In male subjects, the F_50_ of the right lung was significantly greater than that of the left lung at Auscultation Pairs 1, 2, 3, and 4, while vice versa at Auscultation Pair 7. In female subjects, the F_50_ of the right lung was significantly greater than that of the left lung at Auscultation Pairs 1 and 3, while the F_50_ of the right lung was significantly smaller than that of the left lung at Auscultation Pairs 6 and 7.

### 3.7. F_MPD_: Left vs. Right

**Figure 9:**
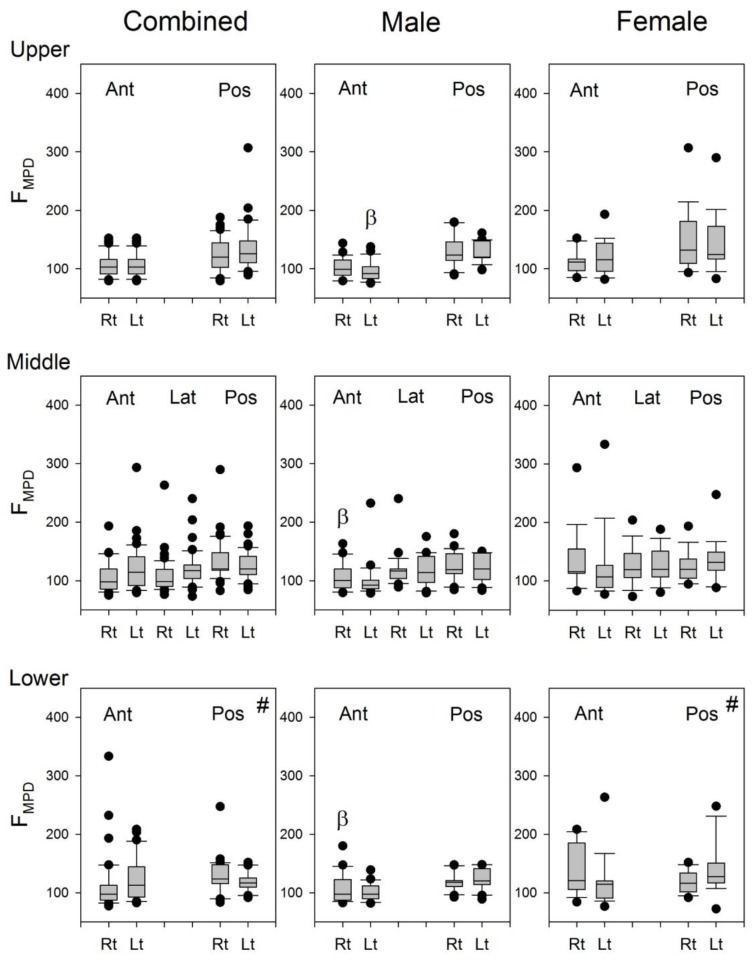
The boxplots of F_MPD_ in the combined group, male group, and female group. #: Three of the 18 female subjects were excluded from the statistics because of excessive artifacts at this site. α: *p* < 0.05 between right and left lungs, β: *p* < 0.05 vs. its counterpart in the female subjects. Ant: anterior; Pos: posterior; Rt: right, Lt: left.

The F_MPD_‘s of the right and left sides were compared and the results are shown in Figure 9. No significant difference in F_MPD_ was found in the combined group, the male group, or the female group. 

## 4. Discussion

This study compared the differences in the spectral characteristics of lung sounds from the left and right lungs. We found that there is asymmetry in the spectral characteristics of lung sounds from the left and right lungs. The P_T_ of lung sounds from the left lung was significantly larger than that from the right lung at most auscultation pairs in combined subjects and in male subjects, but not in female subjects. At the upper posterior auscultation pairs in combined subjects and in male subjects, the situation is reversed in that the P_T_ of lung sounds from the left lung was significantly smaller than that from the right lung. The P_T_ of lung sounds in male subjects was significantly higher than that of female subjects at the three anterior auscultation pairs. The R_I/E_ of lung sounds from the right lung was greater than that from the left lung at auscultation pairs on the anterior chest wall, while it was smaller than that from the left lung at auscultation pairs on the posterior chest wall in combined subjects, and similarly in both male and female subjects. Though the F_MPD_ of lung sounds from the left and right lungs was not significantly different, the F_50_ of lung sounds from the left lung was significantly smaller than that from the right lung at the auscultation site on the anterior and lateral chest walls, while it was significantly larger than that from the right lung at the auscultation site on the posterior chest walls. 

The P_T_ of lung sounds from the left lung was found to be significantly larger than that from the right lung in most auscultation pairs in combined subjects and in male subjects, but not in female subjects. This finding implied that the lung sounds from the left lung is louder than that from the right lung in male subjects, but not in female subjects. Since the left main bronchus makes a sharper angle with the trachea at the carina than the right main bronchus does, a greater turbulence might be created in the left lung when the air is breathed into the lungs. This might account in part for the greater P_T_ of lung sounds from the left lung relative to that from the right lung. Contamination by the heart sound at these auscultation pairs might not be the cause for this difference in P_T_ because this phenomenon was not observed in female subjects. It can also be expected that, for positions near the heart, the P_T_ of lung sounds will be higher in the left lungs. In addition, due to the location of the heart in the left thorax, the two lungs are not the same size, which may have an influence in the P_T_. The smaller volume of the left lung might cause greater turbulence and therefore a greater P**_T_** when the air is breathed into the lungs. These might account for the greater P_T_ of lung sounds in the left lung than that in the right lung. The anatomical differences between males and females may also explain the non-significant difference in the analysis of the females’ P_T_. In contrast, the P_T_ of lung sounds at the left lung was significantly smaller than that at the right lung at the upper posterior auscultation pairs in combined subjects and in male subjects. The cause of this reversal is not clear. Furthermore, contrary to the male subjects, the P_T_ of lung sounds from the left and right lungs was not significantly different at most auscultation sites, except the upper posterior pair in female subjects. The cause of this finding is also not clear. The breasts of the female subjects may interfere with the transmission of the lung sounds to the stethoscope and contribute to the insignificant difference in female’s PT analysis.

The P_T_ of lung sounds in female subjects was significantly smaller than that of male subjects at three anterior auscultation pairs. One reason responsible for this difference might be the increased subcutaneous adipose tissue and the presence of breast on the anterior chest wall in the females because the subcutaneous adipose tissues and the breast might attenuate the lung sounds, leading to decreased total powers of lung sounds at these auscultation sites in female subjects. 

There was a slight negative correlation between the body height and the P_T_ of lung sounds at the upper anterior site of the right lung in male subjects (Table 2). The P_T_ dropped off as the body height increased. According to the Poiseuille’s Law, the resistance of airflow through a tube such as the trachea depends on the length and the radius of the tube. Thus, the negative correlation between the body height and the P_T_ of lung sounds may be explained as being due to the increased resistance in a longer trachea. Because the right main bronchus is longer than the left one and because the upper anterior site of the right lung is close to the trachea, this relation between the body height and the total power of lung sounds was revealed at this auscultation site. There were no significant correlations between body height and the total power of lung sounds at other auscultation sites.

The R_I/E_ of lung sounds from the right lung was greater than that from the left lung at Auscultation Pairs 2 and 3 on the anterior chest wall, while it was smaller than that from the left lung at Auscultation Pair 6 on the posterior chest wall in combined subjects, and similarly in both male and female subjects (Figure 7). It seems that the lung sounds in the inspiratory phase is louder than that in the expiratory phase when auscultated on the right anterior chest wall, while it is louder in the inspiratory phase than in the expiratory phase when auscultated on the left posterior chest wall. The structural differences in the left and right lungs might be the reason for this result. The geometry of airways is not the same in both lungs, especially at the lower parts of the lungs. The right bronchus is longer and thinner, and the tracheobronchial angles at the carina are different between the two lungs. The asymmetric structure in the tracheobroncheal tree might account in part for the difference in the inspiratory/expiratory ratio of P_T_ over the left posterior and right anterior lungs.

The F_50_ at the auscultation site on the anterior and lateral chest walls of the left lung was significantly smaller than that of the right lung, whereas the F_50_ at the auscultation site on the posterior chest walls of the left lung was significantly larger than that of the right lung (Figure 8). The presence of the heart in the lower aspect of the left thorax should have modified the volume and geometry of the lung in the left lower thorax so that the F_50_ at the auscultation site on the posterior chest walls of the left lung was significantly increased, as compared with that on the posterior chest walls of the right lung.

Since the frequency of heart sounds are mostly below 110 Hz [3], it may be speculated that the heart sounds might have some contributions to the calculation of F_50_ and F_MPD_ for the lung sound signals recorded from the left lung. The indifference in the F_MPD_ between the lung sound signals recorded from the right and left chest wall (Figure 9) indicated that the contribution of heart sound to the recorded lung sounds might not be the same as the cause of the significant difference in the F_50_ of lung sounds between the right and left lungs (Figure 8). The difference in the F_50_ of lung sounds between the right and left lungs might be caused by the difference in the volume and structure of the lung. 

## 5. Conclusions

Significant differences in the P_T_, F_MPD_, F_50_, and R_I/E_ between the left and right lungs at some auscultation pairs were observed in this study by using a dual-channel auscultation system. Structural differences between the left and the right lungs, between the female and male subjects, and between anterior and posterior chest walls, might account for the observed differences in the spectral characteristics of lung sounds. The dual-channel auscultation system might be useful for future development of digital stethoscopes and power spectral analysis of lung sounds in patients with various kinds of cardiopulmonary diseases. 

## Figures and Tables

**Table 1:** Basic data of the study subjects.

	Male (*n* = 24)	Female (*n* = 18)
Age (year)	20.8 ± 0.9	20.6 ± 1.1
Weight (kg)	71.1 ± 10.5	51.8 ± 5.3
Height (cm)	173.5 ± 7.4	159.1 ± 5.3
BMI (kg/m^2^)	23.6 ± 3.7	20.4 ± 1.7

Data presented are mean ± SD. BMI: body mass index.

**Table 2:** Linear regression analysis of the correlation between body height and P_T_ of lung sound at each auscultation site for male (*n* = 24) and female (*n* = 18) subjects.

Auscultation Pair	*p* Value			
	Left Lung of Male	Right Lung of Male	Left Lung of Female	Right Lung of Female
1. Upper Anterior	0.118	0.044 *	0.843	0.363
2. Middle Anterior	0.686	0.864	0.570	0.305
3. Lower Anterior	0.584	0.975	0.702	0.803
4. Lateral	0.498	0.641	0.943	0.589
5. Upper Posterior	0.643	0.655	0.345	0.169
6. Middle Posterior	0.595	0.762	0.967	0.607
7. Lower Posterior (#)	0.782	0.810	0.935	0.900

#: Three of the 18 female subjects were excluded from the statistics because of excessive artifacts at this site. * *p* < 0.05.

**Table 3:** Linear regression analysis of the correlation between BMI and the P_T_ at each auscultation site for male (*n* = 23) ^x^ and female (*n* = 18) subjects.

Auscultation Pair	*p*-Value			
	Left Lung of Male	Right Lung of Male	Left Lung of Female	Right Lung of Female
1. Upper Anterior	0.549	0.180	0.815	0.613
2. Middle Anterior	0.627	0.185	0.904	0.820
3. Lower Anterior	0.427	0.291	0.300	0.320
4. Lateral	0.919	0.060	0.513	0.665
5. Upper Posterior	0.348	0.661	0.342	0.845
6. Middle Posterior	0.652	0.777	0.639	0.727
7. Lower Posterior (#)	0.482	0.977	0.228	0.031 *

^x^ One of the 24 male subjects was excluded from the statistics due to excessively high BMI value, 39.6 kg/m^2^, which was 4.35 times the standard deviation from the mean BMI value of male subjects, 23.6 kg/m^2^. # Three of the 18 female subjects were excluded from the statistics because of excessive artifacts at this site. * *p* < 0.05.
